# Comprehensive Succinylome Profiling Reveals the Pivotal Role of Lysine Succinylation in Energy Metabolism and Quorum Sensing of *Staphylococcus epidermidis*

**DOI:** 10.3389/fmicb.2020.632367

**Published:** 2021-02-01

**Authors:** Yiping Zhao, Yang Han, Yuzhe Sun, Zhendong Wei, Jialong Chen, Xueli Niu, Qian An, Li Zhang, Ruiqun Qi, Xinghua Gao

**Affiliations:** ^1^Key Laboratory of Immunodermatology, Department of Dermatology, National Joint Engineering Research Center for Theranostics of Immunological Skin Diseases, Ministry of Health and Ministry of Education, The First Affiliated Hospital of China Medical University, Shenyang, China; ^2^Department of Dermatology, Central Hospital Affiliated to Shenyang Medical College, Shenyang, China; ^3^Department of Dermatology, Dermatological Hospital, Southern Medical University, Guangzhou, China; ^4^Department of Dermatology, The Second Affiliated Hospital of Dalian Medical University, Dalian, China

**Keywords:** post-translational modification, lysine succinylation, *Staphylococcus epidermidis*, energy metabolism, succinyltransferase, desuccinylase, quorum sensing

## Abstract

**Background:**

Lysine succinylation is a newly identified posttranslational modification (PTM), which exists widely from prokaryotes to eukaryotes and participates in various cellular processes, especially in the metabolic processes. *Staphylococcus epidermidis* is a commensal bacterium in the skin, which attracts more attention as a pathogen, especially in immunocompromised patients and neonates by attaching to medical devices and forming biofilms. However, the significance of lysine succinylation in *S. epidermidis* proteins has not been investigated.

**Objectives:**

The purpose of this study was to investigate the physiological and pathological processes of *S. epidermidis* at the level of PTM. Moreover, by analyzing previous succinylome datasets in various organisms, we tried to provide an in-depth understanding of lysine succinylation.

**Methods:**

Using antibody affinity enrichment followed by LC-MS/MS analysis, we examined the succinylome of *S. epidermidis* (ATCC 12228). Then, bioinformatics analysis was performed, including Gene Ontology (GO), KEGG enrichment, motif characterization, secondary structure, protein–protein interaction, and BLAST analysis.

**Results:**

A total of 1557 succinylated lysine sites in 649 proteins were identified in *S. epidermidis* (ATCC 12228). Among these succinylation proteins, GO annotation showed that proteins related to metabolic processes accounted for the most. KEGG pathway characterization indicated that proteins associated with the glycolysis/gluconeogenesis and citrate cycle (TCA cycle) pathway were more likely to be succinylated. Moreover, 13 conserved motifs were identified. The specific motif KsuD was conserved in model prokaryotes and eukaryotes. Succinylated proteins with this motif were highly enriched in the glycolysis/gluconeogenesis pathway. One succinylation site (K144) was identified in S-ribosylhomocysteine lyase, a key enzyme in the quorum sensing system, indicating the regulatory role succinylation may play in bacterial processes. Furthermore, 15 succinyltransferases and 18 desuccinylases (erasers) were predicted in *S. epidermidis* by BLAST analysis.

**Conclusion:**

We performed the first comprehensive profile of succinylation in *S. epidermidis* and illustrated the significant role succinylation may play in energy metabolism, QS system, and other bacterial behaviors. This study may be a fundamental basis to investigate the underlying mechanisms of colonization, virulence, and infection of *S. epidermidis*, as well as provide a new insight into regulatory effects succinylation may lay on metabolic processes (Data are available via ProteomeXchange with identifier PXD022866).

## Introduction

Posttranslational modification (PTM) is a key mechanism in the effective enlargement of the function and diversity of proteins. It mainly occurs on the lysine among 20 amino acids that are the fundamental components of proteins. Because of the nature of lysine, the changes in its charge and structure will affect the composition, activity, and interaction of proteins (Azevedo and Saiardi, 2016; Stram and Payne, 2016; Gao J. et al., 2019). There are more than 620 types of PTMs discovered (Xu et al., 2018), including methylation (Unnikrishnan et al., 2019), acetylation (Barnes et al., 2019), phosphorylation (Pawson and Scott, 2005), ubiquitination (Ronau et al., 2016), crotonylation (Tan et al., 2011), malonylation (Peng et al., 2011), and succinylation (Zhang et al., 2011).

Among all these PTMs, succinylation is a new type of PTM, which was identified by Zhang et al. (2011) using the high throughout HPLC-MS/MS and antibody-based affinity enrichment. The process of succinylation is to transfer the succinyl group from succinyl-CoA to the lysine residue of protein, thus increasing the mass shift of 100.0186 Da and introducing the −2 charge at the same time, finally turning the +1 lysine into −1 succinyllysine. Compared with acetylation or methylation, which adds a group of 42 Da or 14 Da, respectively, succinylation brings more remarkable changes in the structure and charges, which is believed to play a more critical role in physiological and pathological regulation (Sabari et al., 2017). With the rapid development of MS technology, lysine succinylation is revealed to widely exist in prokaryotic and eukaryotic organisms, such as *Escherichia coli*, *Vibrio parahaemolyticus*, *Saccharomyces cerevisiae*, *Trichophyton rubrum*, *Aspergillus flavus*, *Pseudomonas aeruginosa*, *Toxoplasma gondii*, *Dendrobium officinale*, *Mus musculus*, and *Homo sapiens* (Weinert et al., 2013; Li et al., 2014; Pan et al., 2015; Feng et al., 2017; Xu et al., 2017; Gaviard et al., 2018; Ren et al., 2018). It is highly conserved, reversible, and dynamic and plays a vital role in the metabolism in multiple species (Alleyn et al., 2018). In bacteria including *E. coli*, *V. parahaemolyticus*, *Mycobacterium tuberculosis*, *Bacillus subtilis*, *Corynebacterium glutamicum*, *Aeromonas hydrophila*, *P. aeruginosa*, and *Porphyromonas gingivalis*, it is demonstrated that lysine succinylation emerges as a regulator of growth, virulence, antibiotic resistance, and, especially, metabolic processes in bacterial behaviors. Moreover, a plethora of enzymes that play a significant role in multiple pathways are subject to succinylation and conserved among different species, for example, isocitrate dehydrogenase (IDH), carbamoyl phosphate synthase 1 (1), 3-hydroxy-3-methylglutaryl-CoA synthase 2 (HMGCS2), molecular chaperone DnaK, and glyceraldehyde-3-phosphate dehydrogenase (GAPDH) (Weinert et al., 2013; Kosono et al., 2015; Pan et al., 2015; Yang et al., 2015; Mizuno et al., 2016; Gaviard et al., 2018; Wu et al., 2019; Yao et al., 2019). However, the in-depth mechanism and physiological significance of lysine succinylation remain elusive. More research on succinylation should be carried out to elucidate its underlying mechanism and provide a new insight to find possible targets to treat diseases, especially those caused by microorganisms.

*Staphylococcus epidermidis* is a coagulase-negative staphylococcus, which scarcely produces coagulase. Unlike coagulase-positive *Staphylococcus aureus*, which can cause multiple infections of the host, *S. epidermidis* always emerges as a commensal bacterium in the skin and mucous membrane without progressive pathogenicity (Heilmann et al., 2019). However, in recent years, increasing evidence shows that it is an opportunistic bacterium, which can cause infections of the skin, soft tissues, endocardium, and other tissues in patients who are elderly, newborns, or immunocompromised. It is the most frequent causative of nosocomial infection by dwelling in the surgically medical devices (Grice and Segre, 2011; Christensen and Brüggemann, 2014; Argemi et al., 2019). Several cases have been reported of the patients infected with *S. epidermidis*, and the number of these cases increases gradually, which causes a great burden to the public health system (Schoenfelder et al., 2010). This bacterium can form biofilms as virulence to infect the host and escape from the immune elimination. Besides, it is widely present in the skin and mucous membrane. Therefore, avoiding infection with this bacterium during operation or insertion is dramatically complicated (Paharik and Horswill, 2016).

Except for the pathogenicity, it plays various roles in skin homeostasis mechanisms by interacting with the host cells or other skin bacteria. With regard to wound healing, a study revealed that N-formyl methionine peptides, secreted by *S. epidermidis*, are recognized by dendritic cells and presented to CD8 + T cells, subsequently promoting the accumulation of CD8 + T cell and benefitting the wound healing (Linehan et al., 2018). For skin tumors, *S. epidermidis* can suppress the growth of melanoma partially without systematic toxicity by producing 6-N-hydroxyamino-purine, an inhibitor of DNA polymerase activity (Nakatsuji et al., 2018). Additionally, Wang et al. (2017b) reported that *S. epidermidis* generates butyric acid, which promotes the differentiation of adipose-derived stem cells to adipocytes as well as increases the accumulation of cytoplasmic lipid, leading to an increased dermal layer. *S. epidermidis* can release succinic acid that acts on the TLR2 of the host keratinocytes, promoting its production of AMPs (antimicrobial peptides), then competing with *C. acne* (Claudel et al., 2019). Therefore, the abundance of *S. epidermidis* may be a regulatory factor for the colonization of *C. acne* in the skin. Regulating the balance between *S. epidermidis* and C. *acne* may be a new therapy for treating acne. Furthermore, Iwase et al. revealed that *S. epidermidis* secretes a serine protease *Esp*, which can hinder the biofilm formation and nasal colonization of *Staphylococcus aureus*, a severe infectious pathogen of pneumonia, endocarditis, and septicemia. The unknown novel mechanism of *Esp* inhibiting *S. aureus* may be a therapeutic target to prevent the infection caused by S. *aureus* (Iwase et al., 2010). All the above-mentioned studies show that metabolic processes and metabolites may act as essential regulators in physiological and pathological processes of *S. epidermidis*. Still, the interaction between *S. epidermidis* and other skin bacteria as well as its function in skin health remains unclear and deserves more investigation.

Until now, the underlying mechanisms of transforming commensal *S. epidermidis* to an infectious pathogen have not been investigated at the level of PTM. In this study, we conducted the first profile of lysine succinylome in the *S. epidermidis* and elucidated that 1557 lysine sites in 649 proteins were subject to succinylation, which were highly enriched in significant cellular processes including glycolysis/gluconeogenesis, citrate cycle (TCA cycle), pyruvate metabolism, and binding activity. The results proved an essential role succinylation might play in *S. epidermidis.* This study may promote our understanding of bacterial behaviors of *S. epidermidis* at the level of lysine succinylation and provide new insight into the development of effective drugs to treat infections caused by *S. epidermidis*.

## Materials and Methods

### Bacterial Culture

The strain used in this study is *S. epidermidis* (ATCC 12228), which was purchased from the American Type Culture Collection (ATCC). After overnight culture, the *S. epidermidis* (ATCC 12228) cells were inoculated into beef extract-sodium chloride-peptone medium (BSCP) at a ratio of 1:200, and the inoculation was adjusted to make the OD 600 nm 90 μL *S. epidermidis* suspension account for 0.027. Then, mix 50 ml bacterial suspension in a certain concentration with 50 ml BSCP medium and gently shake the mixture at 37°C for 12 h. Centrifugate the suspension at 4000*g* and 4°C for 3 min. Discard the supernatant and wash the remaining debris with PBS three times.

### Protein Extraction, Labeling, Fractionation, and Affinity Enrichment

Samples were ground by liquid nitrogen and sonicated on ice in lysis buffer. The remaining debris was discarded after centrifugation at 20,000*g* and 4°C for 10 min. Finally, the protein was precipitated with cold 15% TCA for 2 h at −20°C. After centrifugation at 4°C for 10 min, the supernatant was removed. The precipitate was washed with precooled acetone three times, dissolved in the buffer (8 M urea, 100 mM TEAB, pH 8.0), and determined with a 2-D Quant kit. For digestion, the protein solution was reduced with 10 mM DTT for 1 h at 37°C and alkylated with 25 mM IAA for 45 min at room temperature in the darkness, then diluted by adding 100 mM TEAB until the urea concentration was less than 2 M. Finally, add the trypsin at the mass ratio of 1:50 trypsin-to-protein for the first digestion overnight and 1:100 trypsin-to-protein for a second 4-h digestion.

After trypsin digestion, the peptides were desalted and further vacuum-dried. Peptides were resuspended in 0.5 M TEAB, and subsequent procedures were conducted according to the manufacturer’s protocol for the 6-plex TMT kit. The samples were fractionated by high pH reversed-phase HPLC using Agilent 300Extend C18 column (5 μm particles, 4.6 mm ID, 250 mm length). Then, the peptides were dried by vacuum centrifugation. To enrich Ksu peptides, tryptic peptides dissolved in NETN buffer (100 mM NaCl, 1 mM EDTA, 50 mM Tris–HCl, 0.5% NP-40, pH 8.0) were incubated with prewashed antibody beads (PTM Biolabs) at 4°C overnight. The beads were washed, and the bound peptides were eluted from the beads. The fractions were combined, vacuum-dried, and cleaned with C18 ZipTips (Millipore) for further LC-MS/MS analysis.

### LC-MS/MS Analysis

The peptides were dissolved in 0.1% FA and then loaded onto a reversed-phase analytical column (Acclaim PepMap RSLC, Thermo Fisher Scientific). The gradient comprised of an increase from 7 to 25% solvent B (0.1% FA in 98% ACN) for 24 min, 25 to 40% for 8 min, and climbing to 80% in 5 min, then holding at 80% for the last 3 min, all at a constant flow rate of 400 nl/min on an EASY-nLC 1000 UPLC system. The peptides were subjected to NSI source followed by tandem mass spectrometry (MS/MS) in Q Exactive Plus (Thermo Fisher Scientific) coupled online to the UPLC. The electrospray voltage applied was 2.0 kV. For full scans, the m/z scan range was 350 to 1800, and intact peptides were detected in the Orbitrap at a resolution of 70,000. Peptides were selected for MS/MS using the NCE setting as 28 and 31; ion fragments were detected in the orbitrap at a resolution of 17,500. A data-dependent procedure was alternated between one MS scan followed by 20 MS/MS scans with a 15.0-s dynamic exclusion. The automatic gain control (AGC) was set at 5E4. The fixed first mass was set as 100 m/z.

### Database Search

The resulting MS/MS data were processed using MaxQuant with an integrated Andromeda search engine (Cox and Mann, 2008). Tandem mass spectra were searched against the *S. epidermidis ATCC12228* database concatenated with the reverse decoy database. Trypsin/P was specified as a cleavage enzyme allowing up to 4 missing cleavages, 5 modifications per peptide. The mass tolerance for precursor ions was set as 20 ppm in the first search and 5 ppm in the main search, and the mass tolerance for fragment ions was set as 0.02 Da. Carbamidomethyl on Cys was specified as fixed modification and oxidation on Met. Succinylation on Lys and acetylation on protein N-terminal were specified as variable modifications. False discovery rate (FDR) thresholds for protein and peptide were specified at 1%, and the minimum score for modified peptides was set >40. The minimum peptide length was set at 7. For the quantification method, TMT 6-plex was selected. The site localization probability was set as >0.75.

### Bioinformatics Analysis

UniProt-GOA, KEGG, and InterPro database were, respectively, used to annotate GO, pathway, and domain information of proteins (Kanehisa et al., 2019; Mitchell et al., 2019; UniProt Consortium, 2019). Webserver software CELLO (v 2.5) was used to predict subcellular localization based on protein sequences. A two-tailed Fisher’s exact test was performed to test the functional enrichment of succinylated protein.

Secondary structure analysis was performed using NetSurfP (v1.0). Only predictions with a minimum probability of 0.5 for one type of secondary structures (coil, α-helix, β-strand) were retained for analysis. The mean secondary structure probabilities of the modified lysine residues were compared with the mean secondary structure probabilities of a control dataset containing all the lysine residues of all the succinylated proteins identified in this study. The *p-*values were calculated by the Wilcoxon test. The protein–protein interaction (PPI) network was conducted by the STRING database (version 11.0) and visualized by Cytoscape software (version 3.8.0) (Doncheva et al., 2019; Szklarczyk et al., 2019).

To predict Ksu motifs, the motif-x was used to analyze peptide sequences that consist of 10 amino acids upstream and downstream of the succinylated lysine site (Chou and Schwartz, 2011). Motif-x analysis parameter: significance threshold was set as 1e-6; min occurrences were set as 20, and the background database was set as all protein sequences of species. All statistical tests and calculations were performed using R (version 3.6.1) (Chan, 2018).

### Data Availability

The raw succinylome datasets generated for this study have been deposited to the PRIDE Archive^1^ (Perez-Riverol et al., 2019) with identifier PXD022866.

## Results

### Identification of 1557 Succinylated Sites in 649 Proteins of *Staphylococcus epidermidis*

We identified a total of 1557 succinylation sites in 649 proteins, in which the FDR was lower than 1%. The distributions of mass error were near zero, most <0.02 (see Supplementary Table 1). Additionally, the length of most succinylated peptides was in the range of 7 to 20. Taken together, both the mass accuracy of MS data and the property of tryptic peptides fit the established standards.

**FIGURE 1:**
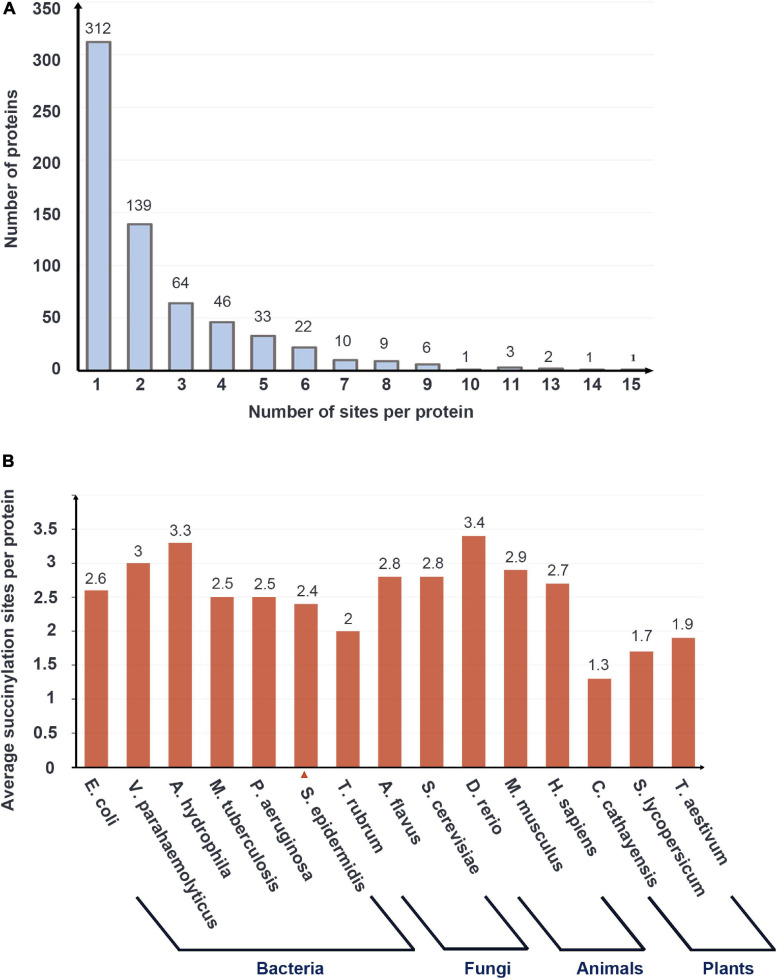
The basic data of LC-MS/MS data and the succinylation profile of *Staphylococcus epidermidis.* **(A)** The number of proteins with the various numbers of succinylated sites. **(B)** Average succinylation sites per protein in bacteria, fungi, animals, and plants.

Of all succinylation proteins identified, 48.1% (312/649) possessed only one modified site, 21.4% (139/649) had two succinylated peptide sites, 9.9% (64/649) contained three sites, and 1.2% (8/649) of modified proteins have 10 or more sites (see Figure 1A). The average degree of succinylation sites per protein was 2.4 (1557/649). Compared with other species including *E. coli* 3.85 (2580/670), *V. parahaemolyticus* 3.0 (1931/642), *A. hydrophila* 3.3 (2174/666), *M. tuberculosis* 2.5 (1545/626), *P. aeruginosa* 2.5 (1520/612), *T. rubrum* 2.0 (569/284), *A. flavus* 2.8 (985/349), *S. cerevisiae* 2.8 (1345/474), *D. rerio* 3.4 (552/164), *H. sapiens* 2.7 (2004/738), *M. musculus* 2.9 (2140/750), *C. cathayensis* 1.3 (259/202), *S. lycopersicum* 1.7 (347/202), and *T. aestivum* 1.9 (330/173) (Weinert et al., 2013; Pan et al., 2015; Yang et al., 2015; Jin and Wu, 2016; Xu et al., 2017; Zhang et al., 2017; Gaviard et al., 2018; Ren et al., 2018; Gao Y. et al., 2019; Yao et al., 2019; Yuan et al., 2019), *S. epidermidis* ranked the last in the bacterial group whose average degree was over 2.5 (see Figure 1B).

Among the total 649 proteins, the most extensively succinylated protein is chaperone protein Dnak, which had 23 modified sites. Foldase protein PrsA, 2-oxoglutarate dehydrogenase E1 component (OGDH), and elongation factor Ts possessed 20, 17, and 17 sites, respectively. Dnak, which is associated with heat shock processes, is reported in the XDR Mtb with 25 succinylated sites (Xie et al., 2015). It is speculated that succinylation may have a significant effect on Dnak activity in response to environmental stimuli.

### Conservation Analysis of Succinylated Proteins in *S. epidermidis* Revealed the Coexisted Similarity and Discrepancy Among Multiple Organisms

**FIGURE 2:**
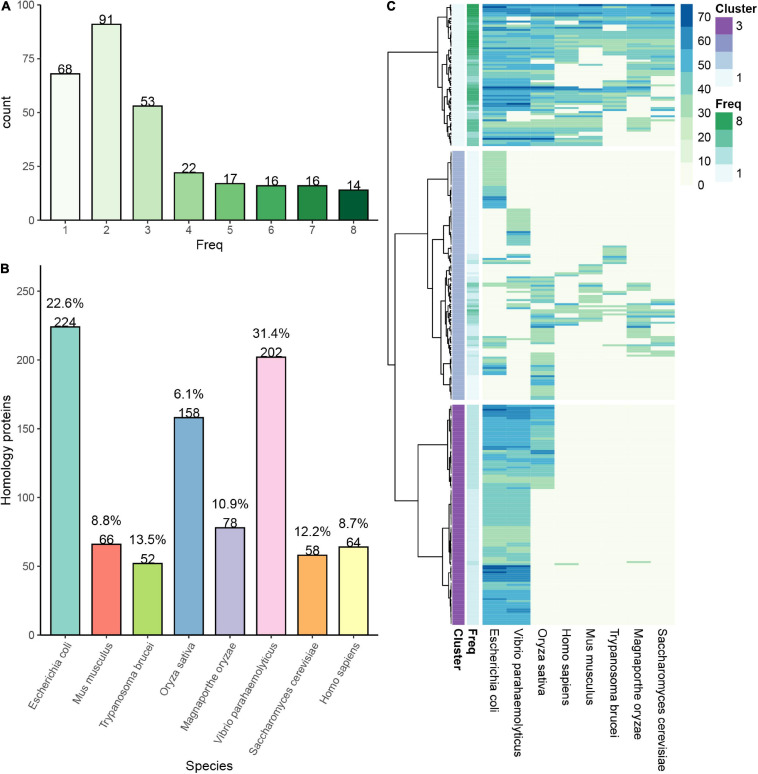
Conservation analysis of identified succinylation proteins in *Staphylococcus epidermidis.* **(A)** The number of proteins in *S. epidermidis* with a different frequency of homology in various organisms. **(B)** The number and percentage of proteins in 8 different species, which are homologous with those in *S. epidermidis.* **(C)** Cluster proteins in *S. epidermidis* are classified into three categories according to their frequency of homology with other 8 species.

A plethora of reports have revealed the high abundance of succinylated proteins from prokaryotes to eukaryotes (Weinert et al., 2013). Investigation of the orthologous proteins in various organisms may elucidate the evolutionary conservation of succinylation. To figure out more features of succinylated events, we performed a comparison of succinylprotein homologs against 8 species with identified succinylomes: *V. parahaemolyticus*, *E. coli*, *Trypanosoma brucei*, *S. cerevisiae*, *Magnaporthe oryzae*, *M. musculus*, *H. sapiens*, and *Oryza sativa* (Weinert et al., 2013; Pan et al., 2015; Zhen et al., 2016; Wang et al., 2019; Zhang et al., 2020). A total of 297 succinylated proteins in *S. epidermidis* were found orthologs in these 8 species. In prokaryotes including *V. parahaemolyticus* and *E. coli*, 202 and 224 succinylated proteins are homologous, which account for 31.42% (202/643) and 22.6% (224/991) of totally identified succinylation events in these two species, respectively. Meanwhile, in eukaryotes like *T. brucei*, *S. cerevisiae*, *M. oryzae*, *M. musculus*, *H. sapiens*, and *O. sativa*, the result was that 52, 58, 78, 66, 64, and 158 succinylation proteins had homologs with *S. epidermidis*, respectively. Moreover, the homologous succinylproteins accounted for 13.51% (52/385), 12.21% (58/475), 10.91% (78/715), 8.79% (66/751), 8.66% (64/739), and 6.09% (158/2594) in terms of corresponding organisms (see Figures 2A,B and Supplementary Table 2). All the above suggested that succinylated proteins were conserved from prokaryotes to eukaryotes. Furthermore, orthologous proteins in prokaryotes accounted more than that in eukaryotes, demonstrating that the number of homologous events of succinylation was higher between two prokaryotes than between prokaryotes and eukaryotes, which was consistent with the evolutionary laws.

Then, we classified the orthologous proteins into four groups according to the number of organisms, which they were homologous in. Succinylproteins that have orthologs in 6∼8 species were grouped into the “highly conserved” category, which accounted for 7.1% (46/649) of all succinylated proteins in S. *epidermidis*. About 14.2% (92/649) proteins were homologous in 3∼5 species, which belonged to the “conserved” category. The “poorly conserved” category included 24.5% (159/649) of total succinylation events, in which homologous succinylproteins were found in 1∼2 species. Finally, proteins subjected to succinylation, which did not have orthologs in other 8 organisms, were clustered to the “novel” category, accounting for 54.2% (352/649) of all succinylated proteins in S. *epidermidis* (see Figure 2C). These results elucidated that despite the conservation of succinylated proteins among species, there still existed unique succinylation events in the distinctive organisms, providing a new insight for investigating specific functions of succinylproteins in particular creatures.

### Functional Annotation and Subcellular Distribution Analysis Showed the Broad Existence of Succinylated Proteins in *S. epidermidis*

**FIGURE 3:**
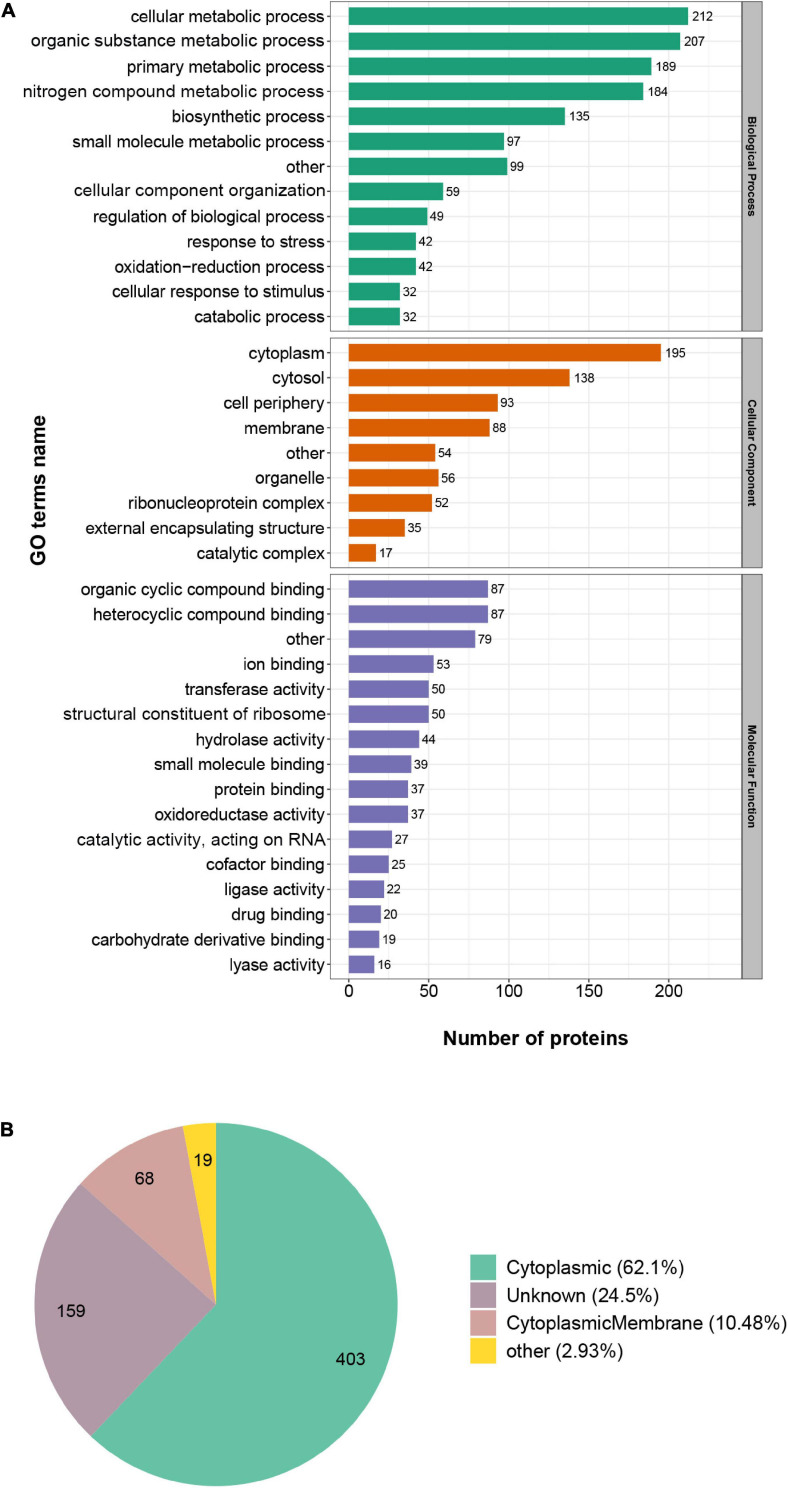
Gene Ontology functional classification of succinylated proteins in *Staphylococcus epidermidis*. **(A)** Classification of succinylated proteins on the basis of biological process (BP), molecular function (MF), and cellular component (CC). **(B)** Classification of succinylated proteins on the basis of subcellular location.

To figure out the role succinylation plays in the cellular processes, we performed the Gene Ontology (GO) functional classification of succinylated proteins in *S. epidermidis* from the perspective of biological process (BP), molecular function (MF), and cellular component (CC) (see Figure 3A and Supplementary Table 3). Based on the biological process, the four largest groups of proteins were all involved in metabolic processes, such as cellular metabolic process (16%), organic substance metabolic process (15%), primary metabolic process (14%), and nitrogen compound metabolic process (13%). The consequence elucidated that succinylation may play a significant role in the metabolic regulation in *S. epidermidis*. Additionally, 3% and 2% succinylated proteins were involved in response to stress and cellular response to the stimulus, respectively, indicating a pivotal mechanism for the survival and adaptation of *S. epidermidis*. With regard to molecular function, proteins involved in organic cyclic compound binding (13%), heterocyclic compound binding (13%), ion binding (8%), and structural constituent of ribosome (7%) were more likely to be succinylated. Moreover, on the basis of cellular component analysis, succinylated proteins preferred to distribute in the cytoplasm (27%), cytosol (19%), cell periphery (13%), and membrane (12%), which was consistent with the consequence of subcellular distribution analysis that cytoplasmic (62%), unknown (25%), and cytoplasmic membrane (10%) proteins accounted for the most among all the identified ones (see Figure 3B). Interestingly, in the cluster of the cellular component, nearly 5% of succinylated proteins were related to external encapsulating structure, which may be an important clue for the virulence of *S. epidermidis*. These results, corresponding to GO annotation and subcellular distribution, elucidated that succinylation existed broadly in nearly all cellular components and played a significant role in multiple processes in the *S. epidermidis*.

### Enrichment Analysis of Succinylated Proteins and Peptides

**FIGURE 4:**
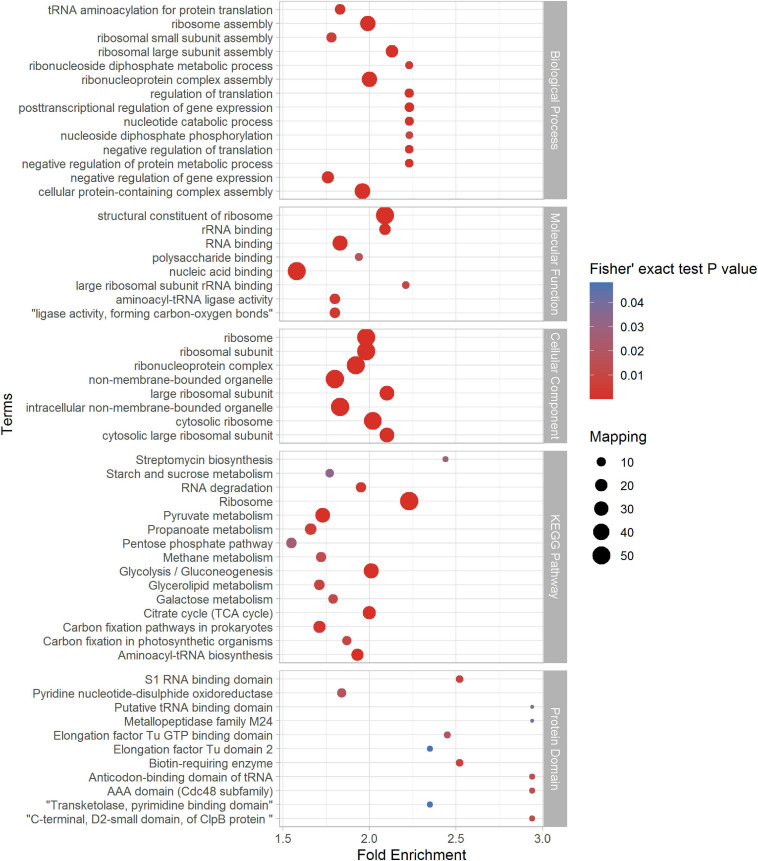
Enrichment analysis of identified succinylproteins in terms of biological process (BP), molecular function (MF), cellular component (CC), KEGG pathway, and protein domain (*p* < 0.05).

In order to figure out the relationship between succinylated proteins and cellular function, we did the enrichment analysis based on GO enrichment, KEGG pathway, and Pfam domain (see Figure 4 and Supplementary Table 4). The consequences showed that in terms of the cellular component in GO enrichment, proteins related to the cytosolic ribosome, ribosome, ribosomal subunit, ribonucleoprotein complex, and intracellular non-membrane-bounded organelle were significantly enriched, indicating the high involvement of succinylation in the ribosomal events. Furthermore, in the light of biological process, succinylated proteins were more likely to enrich in processes including ribonucleoprotein complex assembly, cellular protein-containing complex assembly, ribosome assembly, posttranscriptional regulation of gene expression, and regulation of translation. On the basis of molecular function enrichment, proteins associated with the structural constituent of ribosome, RNA binding, nucleic acid binding, and rRNA binding were highly subjected to succinylation. Moreover, KEGG enrichment indicated that succinylated proteins were more likely to enrich in the pathways related to the ribosome, glycolysis/gluconeogenesis, citrate cycle (TCA cycle), pyruvate metabolism, aminoacyl-tRNA biosynthesis, and glycerolipid metabolism, which was consistent with the results in GO enrichment and aforementioned KEGG pathway analysis of succinylation in other species like *E. coli*, *V. parahaemolyticus*, *H. sapiens*, and others, confirming the important role of succinylated proteins in the metabolism processes. Then, we utilized Pfam to analyze domain features of proteins subjected to succinylation. It is illustrated that biotin-requiring enzyme, S1 RNA-binding domain, AAA domain (Cdc48 subfamily), and anticodon-binding domain of tRNA were significantly enriched.

### Characterization of 13 Conserved Motifs of Succinylation Proteins and Investigation of the Relationship Among Motifs and Functional Processes

**FIGURE 5:**
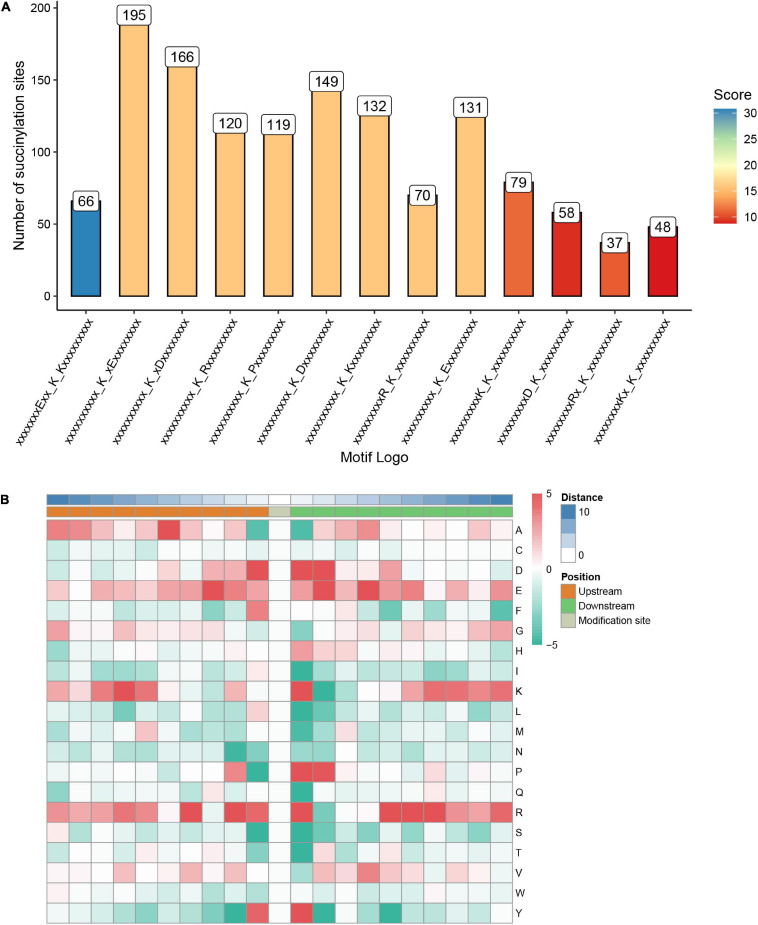
Properties of the succinylated peptides in *Staphylococcus epidermidis.* **(A)** The number of peptides with each of these 13 motifs. **(B)** A heat map of amino acid compositions surrounding the succinylated lysine.

To investigate the nature of succinylated sites in *S. epidermidis*, we use motif-X to characterize the flanking protein sequences of *S. epidermidis* (10 amino acids upstream and downstream of the lysine succinylated site) based on the 1557 succinylated sites identified in this study. Consequently, 13 conserved motifs were identified, whose detailed information was shown below (see Figures 5A,B and Supplementary Table 5). The top five abundant motifs are E^∗∗^KsuK, Ksu^∗^E, Ksu^∗^D, KsuR, and KsuP (Ksu indicates the succinylated lysine sites and ^∗^ represents a random amino acid residue). At the site of + 1 around succinylated lysine, K, R, D, Y, and P were comparably preferred. Also, D, E, and R were most frequent at the −1 site. These results demonstrated that succinylation had a higher tendency to occur around the polar residues (basic or acidic) than the non-polar ones. In the position of + 2, polar acidic residues including D and E were most frequent, which was consistent with the above findings. K was more abundant in the position of −7 and +1 in the identified proteins of *S. epidermidis*. Among these amino acid residues flanking lysine succinylated sites, R was the most frequent one that possessed a high tendency for the −4, +1, +5, +6, and +7 sites around succinyllysine.

**FIGURE 6:**
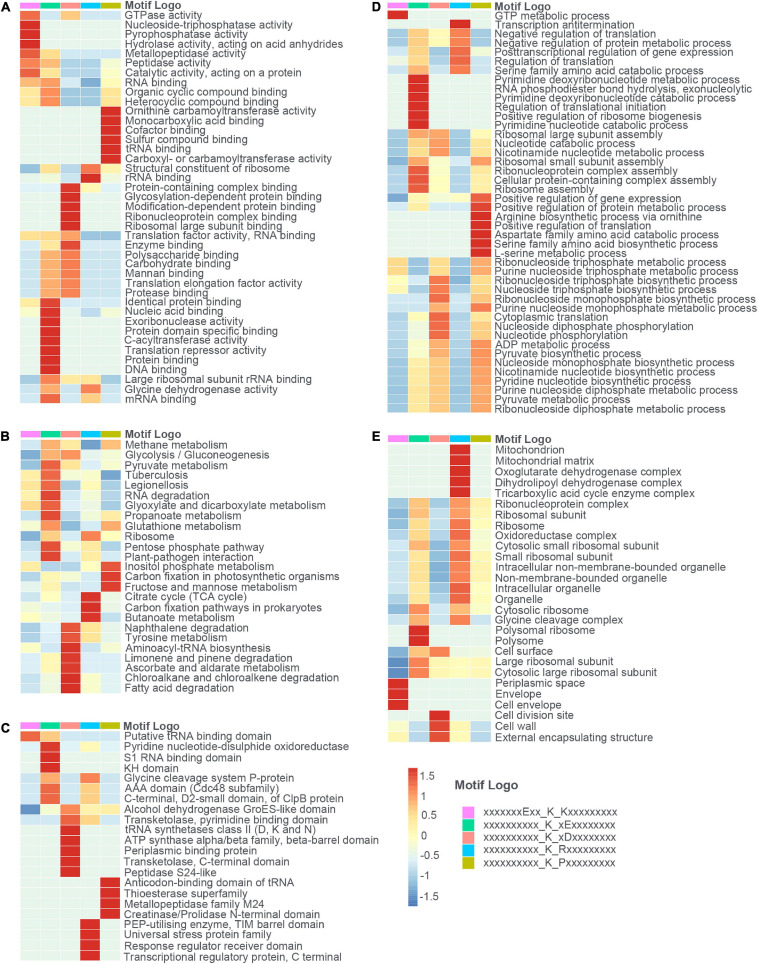
The heat maps showed the enrichment analysis of proteins with each of the five motifs (E**KsuK, Ksu*E, Ksu*D, KsuR, and KsuP) in terms of **(A)** molecular function (MF), **(B)** KEGG pathway, **(C)** protein domain, **(D)** biological process (BP), and **(E)** cellular component (CC).

Aiming to figure out the profound relationship between motifs and cellular functions, we extracted succinylated proteins with these five motifs and clustered them into five groups. Then, we did the GO, KEGG, and domain enrichment analysis of these five clusters (see Figures 6A–E and Supplementary Tables 6–8). The results showed that proteins with specific motifs were associated with corresponding functions, pathways, and domains. For example, proteins enriched in the citrate cycle (TCA cycle), carbon fixation pathways in prokaryotes, and butanoate metabolism possessed the abundant motif of KsuR. A large portion of proteins with the Ksu^∗^D motif was located in the cell wall, cell division site, and external encapsulating structure according to cellular component enrichment analysis. On the basis of molecular function, they were highly associated with binding events like protein-containing complex binding, glycosylation-dependent protein binding, modification-dependent protein binding, and ribonucleoprotein complex binding. Also, proteins with domains like periplasmic binding protein and transketolase, pyrimidine binding domains were enriched. These findings together elucidated the consistency among motif, domain, protein localization, and molecular function. We speculated that there is an underlying relationship between motifs and protein function, which may provide a novel insight for the future prediction of succinylated sites and their roles in cellular processes.

**FIGURE 7:**
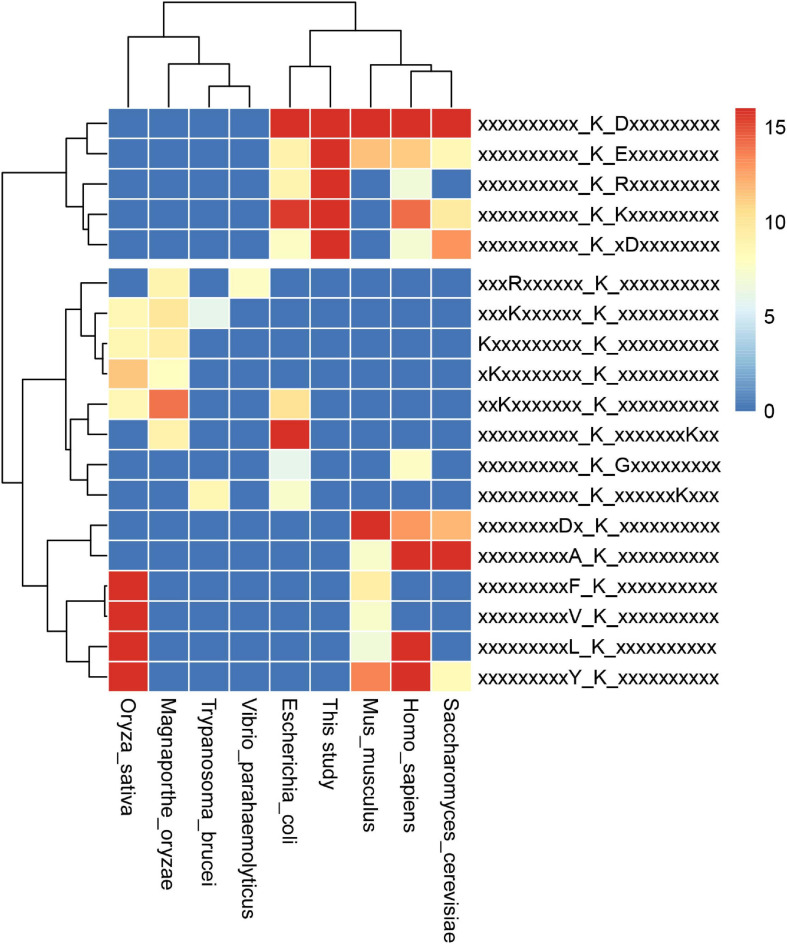
Conservation analysis of identified motifs in the 9 organisms.

Furthermore, we compared the 13 identified motifs in *S. epidermidis* with previously reported succinylomes in *E. coli*, *V. parahaemolyticus*, *T. brucei*, *S. cerevisiae*, *M. oryzae*, *M. musculus*, *H. sapiens*, and *O. sativa* (see Figure 7 and Supplementary Table 9; Weinert et al., 2013; Pan et al., 2015; Zhen et al., 2016; Wang et al., 2019; Zhang et al., 2020). The pattern of K at the + 1 site around succinyllysine was identified in *E. coli*, Hela cells, and yeast. Compared with other motifs reported in previous succinylome studies in various organisms, Ksu^∗^D identified in this investigation was conserved in *E. coli*, Hela cells, and yeast. Meanwhile, KsuD was found in *E. coli*, yeast, Hela cells, and the mouse liver. These results indicated that the nature of succinylated sites was conserved among prokaryotes to eukaryotes. The highly conserved motifs may be unknown targets for the “readers” and “erasers” in the succinylated events, which provided a new insight to investigate the underlying mechanism of succinylation in cellular processes.

**FIGURE 8:**
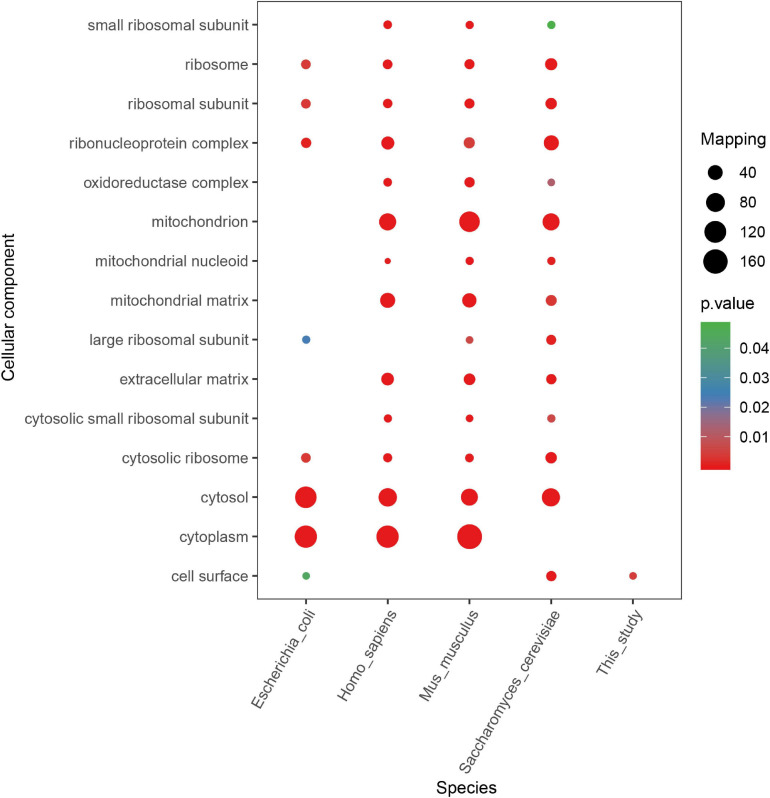
Cellular component enrichment analysis of succinylated proteins with KsuD motif among five organisms (*Escherichia coli*, *Homo sapiens*, *Mus musculus*, *Saccharomyces cerevisiae*, and *Staphylococcus epidermidis*).

**FIGURE 9:**
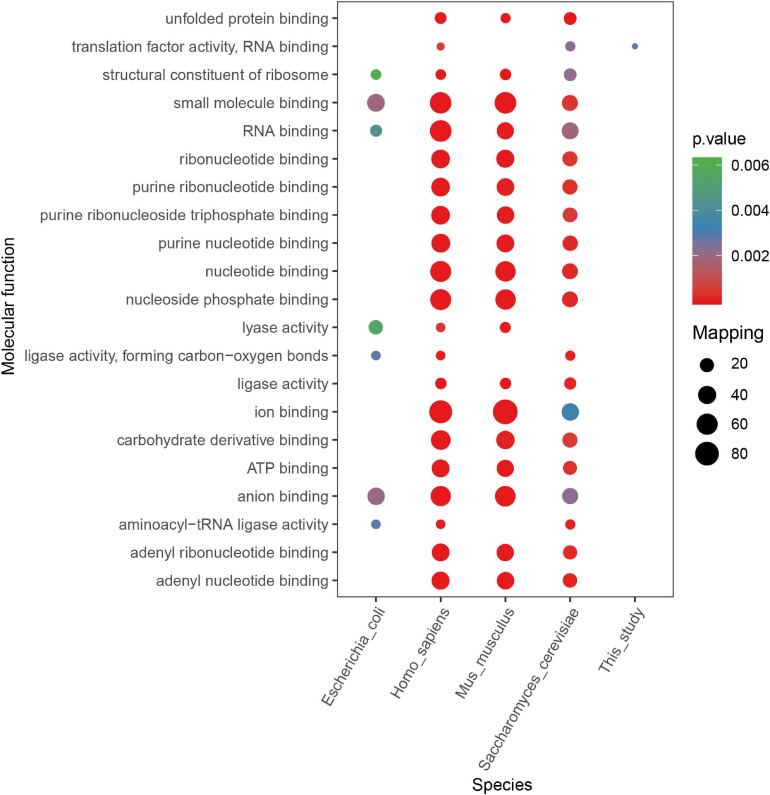
Molecular function enrichment analysis of succinylated proteins with KsuD motif among five organisms (*Escherichia coli*, *Homo sapiens*, *Mus musculus*, *Saccharomyces cerevisiae*, and *Staphylococcus epidermidis*).

**FIGURE 10:**
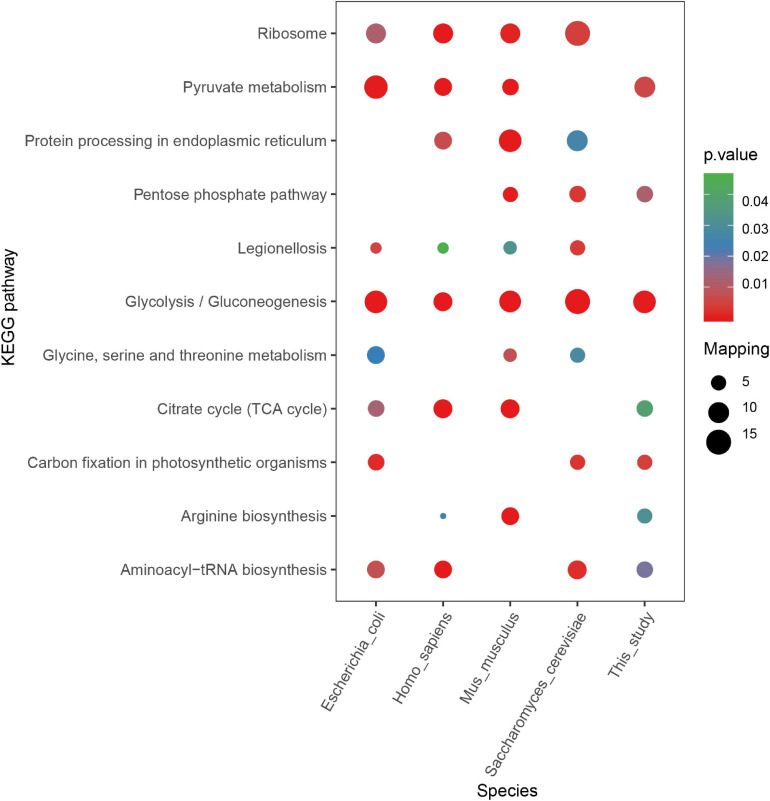
KEGG pathway enrichment analysis of succinylated proteins with the KsuD motif among five organisms (*Escherichia coli*, *Homo sapiens*, *Mus musculus*, *Saccharomyces cerevisiae*, and *Staphylococcus epidermidis*).

According to other reported results, several motifs were conserved in prokaryotes and eukaryotes, which can raise a question that whether these motifs were correlated with a specific function of proteins with them in different species. We did a GO, domain, and KEGG enrichment analysis of proteins with the KsuD motif selected from *E. coli*, *S. cerevisiae*, *M. musculus*, *H. sapiens*, and *S. epidermidis*. It is revealed that proteins located in the cytosol, ribosome, and ribonucleoprotein complex were enriched in four species (*E. coli*, *H. sapiens*, *M. musculus*, and *S. cerevisiae*) based on cellular component enrichment analysis. Moreover, cytoplasmic proteins of *E. coli*, *H. sapiens*, and *M. musculus* had a higher tendency to have a KsuD motif (see Figure 8). According to the result of molecular function enrichment, succinylated proteins associated with binding activities including adenyl nucleotide binding, ATP binding, small molecule binding, nucleoside phosphate binding, and carbohydrate derivative binding were highly enriched in *S. cerevisiae*, *H. sapiens*, and *M. musculus* (see Figure 9). Additionally, succinylated proteins with the KsuD motif had a higher consistency of molecular function in eukaryotes (*S. cerevisiae*, *H. sapiens*, and *M. musculus*) than that in prokaryotes (*E. coli and S. epidermidis*), which is consistent with the laws of evolution. Based on the KEGG enrichment analysis, proteins in the *g*lycolysis/gluconeogenesis pathway were highly enriched in all five organisms, indicating the important role of succinylated proteins in energy metabolism as well as the functional conservation of these succinylation proteins with KsuD motif (see Figure 10).

### Succinylation May Affect the Secondary Structure and Surface Properties of Modified Proteins

**FIGURE 11:**
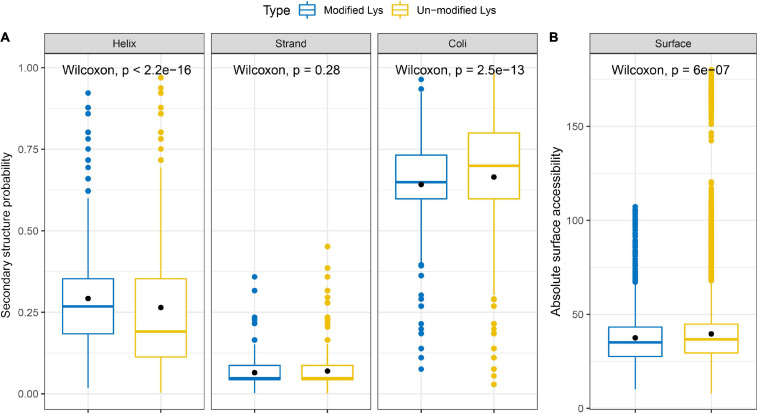
Properties of succinylated sites based on the **(A)** secondary structure probability and **(B)** absolute surface accessibility.

Then, we analyzed the secondary structure of all succinylated proteins in *S. epidermidis* to figure out the relationship between the protein structure and succinylation frequency (see Figure 11A and Supplementary Table 10). The consequences revealed that succinylated events were more abundant in the α-helix (*p* < 2.2e-16) and coils (*p* = 2.5e-13) than that in the β-strand (*p* = 0.28). The percentage of unmodified lysines located in the α-helix of all unmodified peptides was higher than that of succinylated lysines in the α-helix of all succinyllysine residues; however, the results were converse in coli structure. Taken together, it is elucidated that succinylation might change the secondary structure of modified substrates.

Additionally, we investigated the absolute surface accessibility of succinylated lysines (see Figure 11B and Supplementary Table 10). The results demonstrated that succinylated lysine sites were more frequently located on the surface than the unmodified lysines did, leading to the speculation that succinylation tends to the protein surface and succinylated events may alter the surface properties of the modified proteins.

### Succinylation Is a Significant Regulator of Energy Metabolism in *S. epidermidis*

The TCA cycle and glycolysis/gluconeogenesis are key processes to provide energy for organisms, which is essential for survival. A plethora of studies have revealed the preference of lysine succinylation in these metabolic processes (Kosono et al., 2015; Xie et al., 2015; Yang et al., 2015; Feng et al., 2017). In this study, the KEGG pathway enrichment analysis showed that succinylated proteins associated with the TCA cycle and glycolysis/gluconeogenesis pathways were highly enriched. We tried to figure out the regulatory mechanism of lysine succinylation in *S. epidermidis* in terms of metabolic enzymes.

**FIGURE 12:**
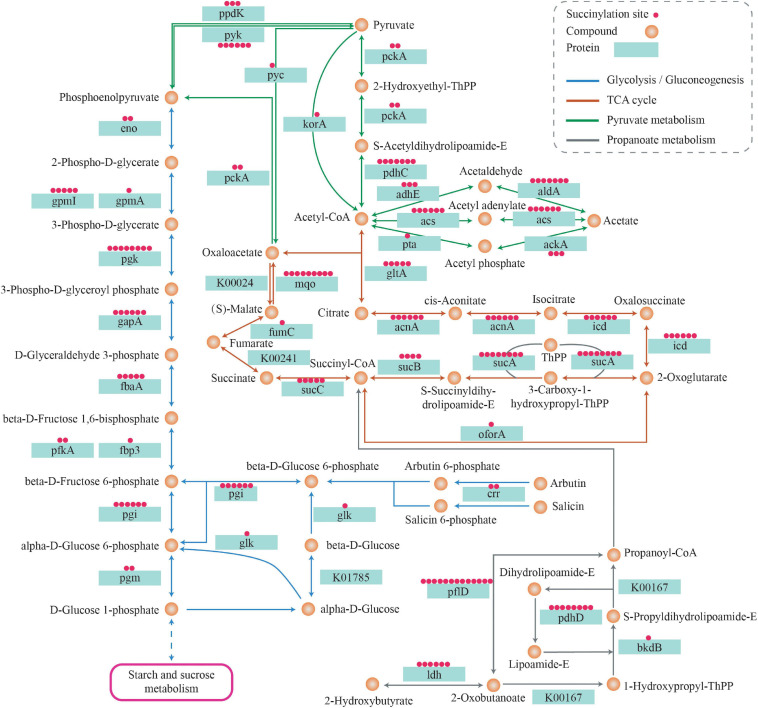
Succinylated proteins related to metabolism pathways.

Nearly all significant enzymes in the Krebs cycle were succinylated in *S. epidermidis*. They were as follows: pyruvate carboxylase, dihydrolipoyl dehydrogenase, aconitate hydratase A, dihydro-lipoyl lysine-residue succinyltransferase component of 2-oxoglutarate dehydrogenase complex, isocitrate dehydrogenase [NADP], succinate-CoA ligase [ADP-forming] subunit beta, citrate synthase, pyruvate dehydrogenase E1 component subunit beta, pyruvate dehydrogenase E1 component subunit alpha, phosphoenolpyruvate carboxykinase [ATP], and 2-oxoglutarate dehydrogenase E1 component. Citrate synthase (SE_1371) converts the oxaloacetate and acetyl-coenzyme A into citrate and coenzyme A, which is the first and rate-limiting step of the Krebs cycle (Li et al., 2016). Five lysine sites of citrate synthase including K64, K117, k262, K357, and K365 were succinylated, indicating the important role of lysine succinylation in the enzymatic regulation. Another pivotal rate-limiting enzyme, isocitrate dehydrogenase (IDH), which catalyzes the oxidation and decarboxylation of isocitrate and produces α-ketoglutarate, carbon dioxide, and NADH + H^+^/NADPH + H^+^ (Tommasini-Ghelfi et al., 2019), was revealed in six succinylated lysine sites: K58, K132, K163, K189, K225, and K262. Meanwhile, in drug-resistant *M. tuberculosis*, a total of 21 succinylated sites were identified on IDH, among which K262 was near the critical catalysis site (K257) (Xie et al., 2015). Furthermore, in *E. coli*, it was demonstrated that the succinylation of K100 and K242 residues of IDH affects the enzyme activity by site mutation analysis (Zhang et al., 2011). Three enzymatic components of α-ketoglutarate dehydrogenase (OGDH) complex—E1 (2-oxoglutarate dehydrogenase), E2 (dihydrolipoamide succinyltransferase), and E3 (dihydrolipoamide dehydrogenase), which together promote the conversion of α-ketoglutarate into succinyl-CoA (Lu et al., 2019), were succinylated at 8, 4, and 7 lysine sites, respectively. Furthermore, five succinylated peptides were also identified in succinyl-CoA synthetases including sucC and sucD that catalyze the reaction of succinyl-CoA hydrolysis and succinate production, which is the only step of substrate-level phosphorylation in the citrate cycle (Huang and Fraser, 2016). Succinyl-CoA is an important donor of the succinyl group for lysine succinylation in proteins. In turn, succinylation affects enzymes that regulate succinyl-CoA production and consumption, indicating a possible cyclic mechanism for regulating succinylation levels in organisms. In addition, proteins associated with the TCA cycle were widely succinylated, which may act as evidence that succinylation plays a pivotal role in regulating energy metabolism in *S. epidermidis* (see Figure 12).

Ten out of ten glycolytic enzymes associated with converting glucose to pyruvate were subject to succinylation. These proteins contained glucokinase, glucose-6-phosphate isomerase, ATP-dependent 6-phosphofructokinase (PFK), fructose-bisphosphate aldolase (FBA), triosephosphate isomerase, glyceraldehyde-3-phosphate dehydrogenase (GAPDH), phosphoglycerate kinase (PGK), 2, 3-bisphosphoglycerate-independent phosphoglycerate mutase (PGPG), enolase (ENO), and pyruvate kinase. Phosphofructokinase catalyzes the conversion of fructose-6-phosphate to fructose-1,6-bisphosphate by transferring a phosphoryl group from ATP, which is the core regulatory step of the glycolytic process (Kim et al., 2017). Two succinylated lysine sites (K77, K212) were identified in PEK, indicating the regulatory role succinylation may play in the glycolysis pathway. Pyruvate kinase, which catalyzes the last step of glycolysis, is one of the three key regulatory enzymes in the glycolysis pathway. It transfers a phosphoryl group from PEP to ADP, then produces pyruvate and ATP (Schormann et al., 2019). In this study, we found six succinylated lysine sites in the pyruvate kinase—K139, K156, K173, K341, K390, and K563. K563 was located in the PEP-utilizing enzyme, a mobile domain at the N-terminus of pyruvate kinase, demonstrating that succinylation of K563 may be a possible regulatory site of the enzyme activity (see Figure 12).

### Protein–Protein Interaction Networks of Succinylated Proteins

**FIGURE 13:**
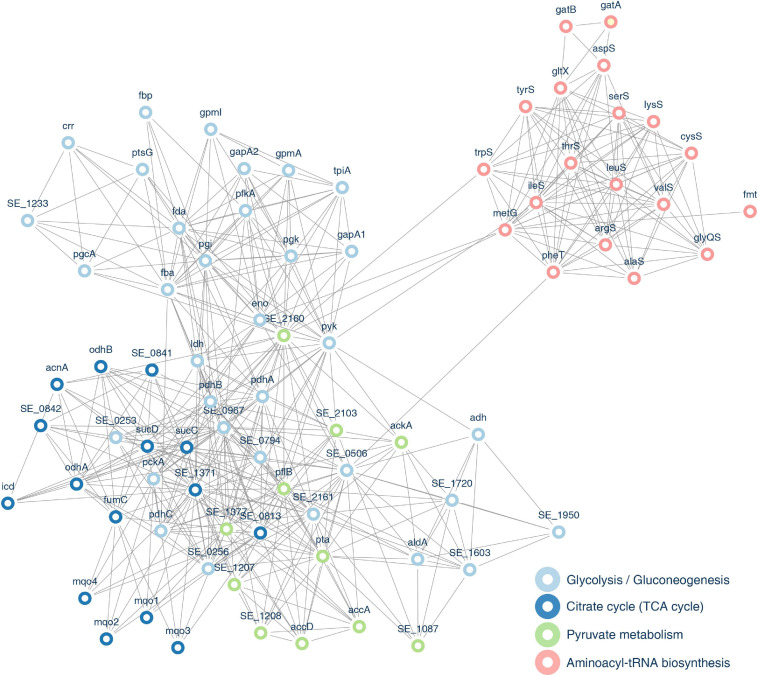
Protein–protein interaction (PPI) network of identified proteins associated with pyruvate metabolism, glycolysis/gluconeogenesis, TCA cycle, and aminoacyl-tRNA biosynthesis pathways.

To figure out the relationship among these identified succinylation proteins, we performed PPI network analysis using the STRING database and Cytoscape software (Doncheva et al., 2019; Szklarczyk et al., 2019). We chose 80 proteins that were related to glycolysis/gluconeogenesis, TCA cycle, pyruvate metabolism, and aminoacyl-tRNA biosynthesis pathways, which were the four most enriched groups in KEGG enrichment analysis (see Figure 13 and Supplementary Table 11). Fourteen succinylated proteins had a degree over 40. Eight of these substrates were associated with the glycolysis/gluconeogenesis pathway, indicating that succinylated proteins in this pathway may play an essential role in biological processes. Four proteins were revealed with the higher degree that was over 50, i.e., SE_0967, SE_1371, SE_2160, and formate acetyltransferase (pflB), among which pflB possessed the most succinylated sites (13 sites), implying that this protein may be a pivotal target to understand the deep mechanisms of succinylation.

## Discussion

Using the MS technology together with the antibody affinity purification method, we performed the first analysis of succinylation profiles in *S. epidermidis* at the level of proteomics. In this study, we revealed a total of 2845 succinylated sites corresponding to 913 proteins in *S. epidermidis*, among which 1557 sites in 649 succinylation proteins were identified after three-time repetition. The different number and functional enrichment of succinylated substrates among various bacterial species may be explained by the discrepancy of inherent succinylation levels of proteins and sites in different bacteria. Furthermore, the divergence of antibody affinity and MS accuracy also account for this difference. Our investigation may enlarge the scale of understanding lysine succinylation in microorganisms, especially *S. epidermidis* which emerged as commensal inhabitants in human skin and mucous membranes as well as opportunistic pathogens of nosocomial infections. It may provide new insights into regulating bacterial survival, invasiveness, and pathogenicity by lysine succinylation.

Lysine succinylation plays an essential role in metabolic processes according to the previous investigation (Colak et al., 2013; Yang et al., 2015). In this study, multiple results illustrated the intensive connections between lysine succinylation and energy metabolism, especially the glycolysis process. For example, the KEGG enrichment analysis showed that proteins associated with the glycolysis/gluconeogenesis pathway had a higher tendency for succinylation. Ten out of ten key enzymes in this pathway were succinylated, especially the pyruvate kinase, which was identified with six modified sites. The result of PPI indicated that eight succinylated proteins associated with glycolysis possessed a degree over 40, the number of which was larger than that of other pathways. All the above demonstrated the significant effect of lysine succinylation on the glycolysis process in *S. epidermidis*. Further study should be conducted to investigate whether lysine succinylation could alter the structure, activity, and interaction of related enzymes.

The quorum system is a commonly existed mechanism in bacteria, which mediates cell-to-cell communication by producing, releasing, accumulating, detecting, and responding to extracellular signaling molecules called autoinducers (AIs) (Rutherford and Bassler, 2012). QS plays a significant role in controlling bacterial behaviors including virulence, biofilm formation, gene expression, and adaptation to a complex environment (Mukherjee and Bassler, 2019). It has been reported that *agr*, a known QS system in *S. epidermidis*, inhibits biofilm formation and alters the structure of biofilms (Le and Otto, 2015; Williams et al., 2019) by regulating biofilm factors including *AtlE* and delta-toxin (Hello et al., 2010; Reiter et al., 2014). Moreover, the LuxS/AI-2-dependent QS system has also been revealed as functional in *S. epidermidis* (Vendeville et al., 2005). LuxS responds to signaling AI-2 and represses virulence and biofilm formation of *S. epidermidis* by mediating transcription of the *ica* genes and production of PIA. LuxS (S-ribosylhomocysteinelyase) is a key enzyme in the QS system, which plays a pivotal role in bacterial behaviors including virulence, pathogenesis, biofilm formation, bioluminescence, and antibiotic resistance (Lewis et al., 2001; Xu et al., 2006). Recently, at the level of PTM, LuxS was identified as a tyrosine kinase phosphorylation site in *V. harveyi* (De Keersmaecker et al., 2006). Furthermore, according to the complete analysis of succinylomes in *A. hydrophila*, two lysine succinylation sites (K23 and K30) were identified in LuxS. Site-specific mutagenesis of K23 and K30 showed that these Ksu sites in LuxS upregulated enzymatic activity and influenced the communication of *A. hydrophila* with other bacteria (Yao et al., 2019). In our study, one succinylated site (K144) was found in LuxS of *S. epidermidis*, which is close to the iron-binding site (C123) and at the terminal of the protein sequence. Further study should focus on the role this succinylated site of LuxS may play in the QS system and bacterial processes, which may be a significant target to understand succinylation and other PTMs.

Succinylation is a newly recognized PTM, whose important regulatory enzymes like desuccinylases and succinyltransferases that possess oppose roles have not been investigated comprehensively yet. KAT2A, KGDHC, and CPT1A are recently identified proteins with succinyltransferase activity in eukaryotes (Gibson et al., 2015; Wang et al., 2017a; Kurmi et al., 2018; Tong et al., 2020). However, succinyltransferases have not been revealed in prokaryotes yet. Lysine acetylation is one of the best-investigated lysine acylations, whose lysine acetyltransferase (KATs) and lysine deacetylases (KDACs) have been identified to possess expanded activities in other acylations including Kpr, Kbu, Kcr, Kbhb, Ksucc, and Kglu (Sabari et al., 2017). For example, KATs p300, a well-known transcription co-activator, has been reported to recognize multiple actyl-CoAs like propionyl-CoA (Chen et al., 2007), butyryl-CoA (Chen et al., 2007), crotonyl-CoA (Sabari et al., 2015), succinyl-CoA (Sabari et al., 2017), glutaryl-CoA (Tan et al., 2014), and β-hydroxybutyryl-CoA (Kaczmarska et al., 2017) and catalyze the corresponding acylation processes. Furthermore, the current structural investigation showed that GCN5 recognizes the common CoA portion of short-chain acyl-CoAs and possesses the identical affinity for acetyl-CoA, propionyl-CoA, and butyryl-CoA (Ringel and Wolberger, 2016; Kollenstart et al., 2019). According to the known discovery, mammalian KATs were categorized into two groups including type A KATs and type B KATs which are located mainly in the nucleus and cytoplasm, respectively (Yang et al., 2011; Li et al., 2020). Type A contains five families including the GNAT, p300/CBP, MYST, Basal TF, and NCoA family (Menzies et al., 2016). Due to the wide range of acyltransferase activities and structural similarity of these acyl-CoAs, we conducted BLAST analysis based on well-known lysine acetyltransferases and revealed 15 proteins in *S. epidermidis* that are homologous with these acetyltransferase families, i.e., GNAT family (Q92830, Q92831, and Q5SQI0), p300/CBP family (Q92793 and Q09472), MYST family (Q92993, Q92794, Q8WYB5, O95251, and Q9H7Z6), NCoA family (Q15788, Q15596, and Q9Y6Q9), and Type B (O14929, Q9H7 × 0). More in-depth studies need to be performed to elucidate whether these proteins have succinyltransferase activities or other enzymes exist that can catalyze succinylation in *S. epidermidis*. It may lay the foundation for further understanding of regulating the dynamic balance of succinylation in bacteria (see Supplementary Table 12).

A total of 18 types of KDACs have been recognized in mammals which are divided into 4 classes. Among them, sir-2 like proteins (SIRT1-7) play a significant role in multiple deacylation processes (Ali et al., 2018). SIRT5 and SIRT7 are two commonly known deacetylases, which have been identified as desuccinylases located mainly in the mitochondria and nucleus, respectively (Osborne et al., 2016). In prokaryotes, sir2-like proteins, i.e., CobB is identified as the first desuccinylase in *E. coli* (Colak et al., 2013). Meanwhile, ScCobB2 in *S. coelicolor* is homologous with SIRT5 and *E. coli* CobB possesses the desuccinylase activity (Zhang et al., 2019). In *S. epidermidis*, we found 18 proteins homologous with HDACs in mammals that may possess deacetylase activity. Among them, Q9NXA8 and Q9NRC8 are in homology with SIRT5 and SIRT7, respectively (see Supplementary Table 12). They are highly possible to desuccinylate modified substrates and regulate bacterial processes in *S. epidermidis*. It is intriguing to explore the possible desuccinylase activity of these proteins and their function in bacterial behaviors of this organism.

In other PTMs like phosphorylation, a complete regulatory system was identified which comprised of “writers” (transfer the acyl groups to targets), “erasers” (remove the acyl groups from the modified substrates), and “readers” (identify the modified peptides and initiate downstream reaction) (Biswas and Rao, 2018). For lysine succinylation, writers (succinyltransferases) and erasers (desuccinylases) were reported in succession. However, the readers have been scarcely reported until now. This may be due to the less specificity of succinylation motifs and fewer datasets of succinylation events. In this study, we tried to figure out the exact motif that existed in most organisms and investigate whether it had functional conservation according to KEGG and GO enrichment analysis. The results revealed that proteins with the same motif (KsuD) in different organisms including *E. coli*, *S. epidermidis*, S. *cerevisiae*, *H. sapiens*, and *M. musculus* were highly enriched in the glycolysis/gluconeogenesis pathway and pyruvate metabolism pathway based on KEGG enrichment analysis. Accordingly, it is possible to hypothesize that the KsuD motif could be a clue to figure out the “reader” that can recognize succinylated events and initiate further reactions. Otherwise, one motif in five organisms is too little to elucidate a common phenomenon or principle. Further study should be undertaken to include more species and motifs and investigate the stable pattern of succinylated events.

There are a couple of limitations in the present study. Firstly, experimental verification like Co-IP and site mutation should be carried out in further studies. Secondly, whether or not various PTMs such as acetylation, malonylation, and crotonylation in adjacent sites have different effects should be investigated deeply in the next project. Thirdly, the enzyme activity of predicted succinyltransferases and desuccinylases in *S. epidermidis* should be verified later. We hope to take this research as a starting point to further explore the physiological and pathological mechanisms of *S. epidermidis* at the level of PTM.

## Conclusion

In this study, we elucidated 649 proteins with 1557 succinylated lysine sites in *S. epidermidis* using antibody affinity purification and MS technology, which is the first comprehensive succinylation profile in this organism. GO annotation, KEGG enrichment, and PPI network showed strong connections between lysine succinylation and metabolic processes. We identified 13 conserved motifs and tried to figure out the functional and pattern relativity from the perspective of motifs (KsuD), which provides a new insight for investigating features and regulatory factors, i.e., “readers” of succinylation; 15 succinyltransferases and 18 desuccinylases were predicted that could be pivotal regulators of succinylated events in this organism. Proteins associated with survival, metabolism, virulence, and cell-to-cell communication were succinylated in *S. epidermidis*, indicating the potential role succinylation may play in bacterial behaviors of this species. This study lays the foundation to deepen the understanding of succinylated events and provide a promising reference to develop therapeutic targets against infections caused by *S. epidermidis*.

## Data Availability Statement

The proteomic data generated for this study (ID: 632367) have been deposited to the PRIDE Archive (https://www.ebi.ac.uk/pride) with identifier PXD022866.

## Author Contributions

YZ and XG conceived the project. YH, RQ, and YZ designed the study. YH and YS performed the experiments. YZ, ZW, and JC involved in the figure preparation. YZ, XN, QA, and LZ participated in the data analysis. YZ wrote the manuscript. YS checked the manuscript. XG corrected and evaluated the manuscript. All authors contributed to the article and approved the submitted version.

## Conflict of Interest

The authors declare that the research was conducted in the absence of any commercial or financial relationships that could be construed as a potential conflict of interest.

## Footnotes

1 https://www.ebi.ac.uk/pride

## References

[B1] Ali I. Conrad R. J. Verdin E. Ott M. (2018). Lysine Acetylation goes global: from epigenetics to metabolism and therapeutics. *Chem. Rev.* 118 1216–1252. 10.1021/acs.chemrev.7b00181 29405707 PMC6609103

[B2] Alleyn M. Breitzig M. Lockey R. Kolliputi N. (2018). The dawn of Succinylation: a posttranslational modification. *Am. J. Physiol. Cell Physiol.* 314 C228–C232. 10.1152/ajpcell.00148.2017 29167150 PMC5866436

[B3] Argemi X. Hansmann Y. Prola K. Prévost G. (2019). Coagulase-negative *Staphylococci pathogenomics*. *Int. J. Mol. Sci.* 20:1215. 10.3390/ijms20051215 30862021 PMC6429511

[B4] Azevedo C. Saiardi A. (2016). Why always lysine? The ongoing tale of one of the most modified amino acids. *Adv. Biol. Regul.* 60 144–150. 10.1016/j.jbior.2015.09.008 26482291

[B5] Barnes C. E. English D. M. Cowley S. M. (2019). Acetylation & Co: an expanding repertoire of histone acylations regulates chromatin and transcription. *Essays Biochem.* 63 97–107. 10.1042/EBC20180061 30940741 PMC6484784

[B6] Biswas S. Rao C. M. (2018). Epigenetic tools (The writers, the readers and the erasers) and their implications in cancer therapy. *Eur. J. Pharmacol.* 837 8–24. 10.1016/j.ejphar.2018.08.021 30125562

[B7] Chan B. K. C. (2018). Data analysis using R programming. *Adv. Exp. Med. Biol.* 1082 47–122. 10.1007/978-3-319-93791-5_2 30357717

[B8] Chen Y. Sprung R. Tang Y. Ball H. Sangras B. Kim S. C. (2007). Lysine propionylation and butyrylation are novel post-translational modifications in histones. *Mol. Cell. Proteom.* 6 812–819. 10.1074/mcp.M700021-MCP200 17267393 PMC2911958

[B9] Chou M. F. Schwartz D. (2011). Biological sequence motif discovery using motif-x. *Curr. Protoc. Bioinform.* 13:Unit 13.15-24. 10.1002/0471250953.bi1315s35 21901740

[B10] Christensen G. J. M. Brüggemann H. (2014). Bacterial skin commensals and their role as host guardians. *Benef. Microb.* 5 201–215. 10.3920/BM2012.0062 24322878

[B11] Claudel J.-P. Auffret N. Leccia M.-T. Poli F. Corvec S. Dréno B. (2019). *Staphylococcus epidermidis*: a potential new player in the physiopathology of acne? *Dermatology* 235 287–294. 10.1159/000499858 31112983

[B12] Colak G. Xie Z. Zhu A. Y. Dai L. Lu Z. Zhang Y. (2013). Identification of lysine Succinylation substrates and the Succinylation regulatory enzyme CobB in *Escherichia coli*. *Mol. Cell Proteom.* 12 3509–3520. 10.1074/mcp.M113.031567 24176774 PMC3861704

[B13] Cox J. Mann M. (2008). MaxQuant enables high peptide identification rates, individualized p.p.b.-range mass accuracies and proteome-wide protein quantification. *Nat. Biotechnol.* 26 1367–1372. 10.1038/nbt.1511 19029910

[B14] De Keersmaecker S. C. J. Sonck K. Vanderleyden J. (2006). Let LuxS speak up in AI-2 signaling. *Trends Microbiol.* 14 114–119. 10.1016/j.tim.2006.01.003 16459080

[B15] Doncheva N. T. Morris J. H. Gorodkin J. Jensen L. J. (2019). Cytoscape StringApp: network analysis and visualization of proteomics data. *J. Proteome Res.* 18 623–632. 10.1021/acs.jproteome.8b00702 30450911 PMC6800166

[B16] Feng S. Jiao K. Guo H. Jiang M. Hao J. Wang H. (2017). Succinyl-proteome profiling of *Dendrobium officinale*, an important traditional Chinese orchid herb, revealed involvement of Succinylation in the glycolysis pathway. *BMC Genom.* 18:598. 10.1186/s12864-017-3978-x 28797234 PMC5553593

[B17] Gao J. Shao K. Chen X. Li Z. Liu Z. Yu Z. (2019). The involvement of post-translational modifications in cardiovascular pathologies: focus on SUMOylation, neddylation, Succinylation, and prenylation. *J. Mol. Cell. Cardiol.* 138 49–58. 10.1016/j.yjmcc.2019.11.146 31751566

[B18] Gao Y. Lee H. Kwon O. K. Tan M. Kim K.-T. Lee S. (2019). Global proteomic analysis of lysine Succinylation in Zebrafish (*Danio rerio*). *J. Proteome Res.* 18 3762–3769. 10.1021/acs.jproteome.9b00462 31483678

[B19] Gaviard C. Broutin I. Cosette P. Dé E. Jouenne T. Hardouin J. (2018). Lysine Succinylation and Acetylation in *Pseudomonas aeruginosa*. *J. Proteome Res.* 17 2449–2459. 10.1021/acs.jproteome.8b00210 29770699

[B20] Gibson G. E. Xu H. Chen H.-L. Chen W. Denton T. T. Zhang S. (2015). Alpha-ketoglutarate dehydrogenase complex-dependent Succinylation of proteins in neurons and neuronal cell lines. *J. Neurochem.* 134 86–96. 10.1111/jnc.13096 25772995 PMC4472501

[B21] Grice E. A. Segre J. A. (2011). The skin microbiome. *Nat. Revi. Microbiol.* 9 244–253. 10.1038/nrmicro2537 21407241 PMC3535073

[B22] Heilmann C. Ziebuhr W. Becker K. (2019). Are coagulase-negative staphylococci virulent? *Clin. Microbiol. Infect.* 25 1071–1080. 10.1016/j.cmi.2018.11.012 30502487

[B23] Hello M. Caroff N. Jacqueline C. Caillon J. Potel G. Batard E. (2010). Influence of the AtlE autolysin on the activity of cell wall-active agents against *Staphylococcus epidermidis*. *Intern. J. Antimicrob. Agents* 35 204–206. 10.1016/j.ijantimicag.2009.09.026 19942412

[B24] Huang J. Fraser M. E. (2016). Structural basis for the binding of succinate to succinyl-CoA synthetase. *Acta Crystallogr. D Struct. Biol.* 72 912–921. 10.1107/S2059798316010044 27487822

[B25] Iwase T. Uehara Y. Shinji H. Tajima A. Seo H. Takada K. (2010). *Staphylococcus epidermidis* Esp inhibits *Staphylococcus aureus* biofilm formation and nasal colonization. *Nature* 465 346–349. 10.1038/nature09074 20485435

[B26] Jin W. Wu F. (2016). Proteome-wide identification of lysine Succinylation in the Proteins of Tomato (*Solanum lycopersicum*). *PLoS One* 11:e0147586. 10.1371/journal.pone.0147586 26828863 PMC4734689

[B27] Kaczmarska Z. Ortega E. Goudarzi A. Huang H. Kim S. Márquez J. A. (2017). Structure of p300 in complex with acyl-CoA variants. *Nat. Chem. Biol.* 13 21–29. 10.1038/nchembio.2217 27820805 PMC5757799

[B28] Kanehisa M. Sato Y. Furumichi M. Morishima K. Tanabe M. (2019). New approach for understanding genome variations in KEGG. *Nucleic Acids Res.* 47 D590–D595. 10.1093/nar/gky962 30321428 PMC6324070

[B29] Kim N. H. Cha Y. H. Lee J. Lee S.-H. Yang J. H. Yun J. S. (2017). Snail reprograms glucose metabolism by repressing phosphofructokinase PFKP allowing cancer cell survival under metabolic stress. *Nat. Commun.* 8:14374. 10.1038/ncomms14374 28176759 PMC5309788

[B30] Kollenstart L. de Groot A. J. L. Janssen G. M. C. Cheng X. Vreeken K. Martino F. (2019). Gcn5 and Esa1 function as histone crotonyltransferases to regulate crotonylation-dependent transcription. *J. Biol. Chem.* 294 20122–20134. 10.1074/jbc.RA119.010302 31699900 PMC6937567

[B31] Kosono S. Tamura M. Suzuki S. Kawamura Y. Yoshida A. Nishiyama M. (2015). Changes in the acetylome and succinylome of *Bacillus subtilis* in response to carbon source. *PLoS One* 10:e0131169. 10.1371/journal.pone.0131169 26098117 PMC4476798

[B32] Kurmi K. Hitosugi S. Wiese E. K. Boakye-Agyeman F. Gonsalves W. I. Lou Z. (2018). Carnitine palmitoyltransferase 1A has a lysine succinyltransferase activity. *Cell Rep.* 22 1365–1373. 10.1016/j.celrep.2018.01.030 29425493 PMC5826573

[B33] Le K. Y. Otto M. (2015). Quorum-sensing regulation in staphylococci-an overview. *Front. Microbiol.* 6:1174. 10.3389/fmicb.2015.01174 26579084 PMC4621875

[B34] Lewis H. A. Furlong E. B. Laubert B. Eroshkina G. A. Batiyenko Y. Adams J. M. (2001). A Structural genomics approach to the study of quorum sensing: crystal structures of three LuxS orthologs. *Structure* 9 527–537. 10.1016/S0969-2126(01)00613-X 11435117

[B35] Li P. Ge J. Li H. (2020). Lysine acetyltransferases and lysine deacetylases as targets for cardiovascular disease. *Nat. Rev. Cardiol.* 17 96–115. 10.1038/s41569-019-0235-9 31350538

[B36] Li X. Hu X. Wan Y. Xie G. Li X. Chen D. (2014). Systematic identification of the lysine Succinylation in the protozoan parasite *Toxoplasma gondii*. *J. Proteome Res.* 13 6087–6095. 10.1021/pr500992r 25377623

[B37] Li Y. Wang H. Dai F. Li P. Jin X. Huang Y. (2016). Label-free fluorescent enzymatic assay of citrate synthase by CoA-Au(I) co-ordination polymer and its application in a multi-enzyme logic gate cascade. *Biosens. Bioelectron.* 86 1038–1046. 10.1016/j.bios.2016.07.107 27501341

[B38] Linehan J. L. Harrison O. J. Han S.-J. Byrd A. L. Vujkovic-Cvijin I. Villarino A. V. (2018). Non-classical immunity controls microbiota impact on skin immunity and tissue repair. *Cell* 172 784–796.e18. 10.1016/j.cell.2017.12.033 29358051 PMC6034182

[B39] Lu X. Yang P. Zhao X. Jiang M. Hu S. Ouyang Y. (2019). OGDH mediates the inhibition of SIRT5 on cell proliferation and migration of gastric cancer. *Exper. Cell Res.* 382:111483. 10.1016/j.yexcr.2019.06.028 31247190

[B40] Menzies K. J. Zhang H. Katsyuba E. Auwerx J. (2016). Protein Acetylation in metabolism - metabolites and cofactors. *Nat. Rev. Endocrinol.* 12 43–60. 10.1038/nrendo.2015.181 26503676

[B41] Mitchell A. L. Attwood T. K. Babbitt P. C. Blum M. Bork P. Bridge A. (2019). InterPro in 2019: improving coverage, classification and access to protein sequence annotations. *Nucleic Acids Res.* 47 D351–D360. 10.1093/nar/gky1100 30398656 PMC6323941

[B42] Mizuno Y. Nagano-Shoji M. Kubo S. Kawamura Y. Yoshida A. Kawasaki H. (2016). Altered Acetylation and Succinylation profiles in *Corynebacterium glutamicum* in response to conditions inducing glutamate overproduction. *Microbiologyopen* 5 152–173. 10.1002/mbo3.320 26663479 PMC4767432

[B43] Mukherjee S. Bassler B. L. (2019). Bacterial quorum sensing in complex and dynamically changing environments. *Nat. Rev. Microbiol.* 17 371–382. 10.1038/s41579-019-0186-5 30944413 PMC6615036

[B44] Nakatsuji T. Chen T. H. Butcher A. M. Trzoss L. L. Nam S.-J. Shirakawa K. T. (2018). A commensal strain of *Staphylococcus epidermidis* protects against skin neoplasia. *Sci. Adv.* 4:eaao4502. 10.1126/sciadv.aao4502 29507878 PMC5834004

[B45] Osborne B. Bentley N. L. Montgomery M. K. Turner N. (2016). The role of mitochondrial sirtuins in health and disease. *Free Rad. Biol. Med.* 100 164–174. 10.1016/j.freeradbiomed.2016.04.197 27164052

[B46] Paharik A. E. Horswill A. R. (2016). The *Staphylococcal* biofilm: adhesins, regulation, and host response. *Microbiol. Spectr.* 4:10.1128/microbiolsec.VMBF-0022-2015. 10.1128/microbiolspec.VMBF-0022-2015 27227309 PMC4887152

[B47] Pan J. Chen R. Li C. Li W. Ye Z. (2015). Global analysis of protein lysine Succinylation profiles and their overlap with lysine Acetylation in the marine *Bacterium vibrio* parahemolyticus. *J. Proteome Res.* 14 4309–4318. 10.1021/acs.jproteome.5b00485 26369940

[B48] Pawson T. Scott J. D. (2005). Protein phosphorylation in signaling–50 years and counting. *Trends Biochem. Sci.* 30 286–290. 10.1016/j.tibs.2005.04.013 15950870

[B49] Peng C. Lu Z. Xie Z. Cheng Z. Chen Y. Tan M. (2011). The first identification of lysine malonylation substrates and its regulatory enzyme. *Mol. Cell Proteom.* 10:M111.012658. 10.1074/mcp.M111.012658 21908771 PMC3237090

[B50] Perez-Riverol Y. Csordas A. Bai J. Bernal-Llinares M. Hewapathirana S. Kundu D. J., et al. (2019). The PRIDE database and related tools and resources in 2019: improving support for quantification data. *Nucleic Acids Res.* 47 D442–D450. 10.1093/nar/gky1106 30395289 PMC6323896

[B51] Reiter K. C. Sant’Anna F. H. d’Azevedo P. A. (2014). Upregulation of icaA, atlE and aap genes by linezolid but not vancomycin in *Staphylococcus epidermidis* RP62A biofilms. *Intern. J. Antimicrob. Agents* 43 248–253. 10.1016/j.ijantimicag.2013.12.003 24389080

[B52] Ren S. Yang M. Yue Y. Ge F. Li Y. Guo X. (2018). Lysine Succinylation contributes to Aflatoxin production and pathogenicity in *Aspergillus flavus*. *Mol. Cell Proteom.* 17 457–471. 10.1074/mcp.RA117.000393 29298838 PMC5836371

[B53] Ringel A. E. Wolberger C. (2016). Structural basis for acyl-group discrimination by human Gcn5L2. *Acta Crystallograph. Sect. D Struct. Biol.* 72 841–848. 10.1107/S2059798316007907 27377381 PMC4932917

[B54] Ronau J. A. Beckmann J. F. Hochstrasser M. (2016). Substrate specificity of the ubiquitin and Ubl proteases. *Cell Res.* 26 441–456. 10.1038/cr.2016.38 27012468 PMC4822132

[B55] Rutherford S. T. Bassler B. L. (2012). Bacterial quorum sensing: its role in virulence and possibilities for its control. *Cold Spring Harb. Perspect. Med.* 2:a012427. 10.1101/cshperspect.a012427 23125205 PMC3543102

[B56] Sabari B. R. Tang Z. Huang H. Yong-Gonzalez V. Molina H. Kong H. E. (2015). Intracellular crotonyl-CoA stimulates transcription through p300-catalyzed histone crotonylation. *Mol. Cell* 58 203–215. 10.1016/j.molcel.2015.02.029 25818647 PMC4501262

[B57] Sabari B. R. Zhang D. Allis C. D. Zhao Y. (2017). Metabolic regulation of gene expression through histone acylations. *Nat. Rev. Mol. Cell Biol.* 18 90–101. 10.1038/nrm.2016.140 27924077 PMC5320945

[B58] Schoenfelder S. M. K. Lange C. Eckart M. Hennig S. Kozytska S. Ziebuhr W. (2010). Success through diversity - how *Staphylococcus epidermidis* establishes as a nosocomial pathogen. *Int. J. Med. Microbiol.* 300 380–386. 10.1016/j.ijmm.2010.04.011 20451447

[B59] Schormann N. Hayden K. L. Lee P. Banerjee S. Chattopadhyay D. (2019). An overview of structure, function, and regulation of *Pyruvate kinases*. *Protein Sci. A Public. Protein Soc.* 28 1771–1784. 10.1002/pro.3691 31342570 PMC6739817

[B60] Stram A. R. Payne R. M. (2016). Post-translational modifications in mitochondria: protein signaling in the powerhouse. *Cell. Mol. Life Sci.* 73 4063–4073. 10.1007/s00018-016-2280-4 27233499 PMC5045789

[B61] Szklarczyk D. Gable A. L. Lyon D. Junge A. Wyder S. Huerta-Cepas J. (2019). STRING v11: protein-protein association networks with increased coverage, supporting functional discovery in genome-wide experimental datasets. *Nucleic Acids Res.* 47 D607–D613. 10.1093/nar/gky1131 30476243 PMC6323986

[B62] Tan M. Luo H. Lee S. Jin F. Yang J. S. Montellier E. (2011). Identification of 67 histone marks and histone lysine crotonylation as a new type of histone modification. *Cell* 146 1016–1028. 10.1016/j.cell.2011.08.008 21925322 PMC3176443

[B63] Tan M. Peng C. Anderson K. A. Chhoy P. Xie Z. Dai L. (2014). Lysine glutarylation is a protein posttranslational modification regulated by SIRT5. *Cell Metab.* 19 605–617. 10.1016/j.cmet.2014.03.014 24703693 PMC4108075

[B64] Tommasini-Ghelfi S. Murnan K. Kouri F. M. Mahajan A. S. May J. L. Stegh A. H. (2019). Cancer-associated mutation and beyond: the emerging biology of isocitrate dehydrogenases in human disease. *Sci. Adv.* 5:eaaw4543. 10.1126/sciadv.aaw4543 31131326 PMC6530995

[B65] Tong Y. Guo D. Yan D. Ma C. Shao F. Wang Y. (2020). KAT2A succinyltransferase activity-mediated 14-3-3ζ upregulation promotes β-catenin stabilization-dependent glycolysis and proliferation of pancreatic carcinoma cells. *Cancer Lett.* 469 1–10. 10.1016/j.canlet.2019.09.015 31610265

[B66] UniProt Consortium (2019). UniProt: a worldwide hub of protein knowledge. *Nucleic Acids Res.* 47 D506–D515. 10.1093/nar/gky1049 30395287 PMC6323992

[B67] Unnikrishnan A. Freeman W. M. Jackson J. Wren J. D. Porter H. Richardson A. (2019). The role of DNA methylation in epigenetics of aging. *Pharmacol. Ther.* 195 172–185. 10.1016/j.pharmthera.2018.11.001 30419258 PMC6397707

[B68] Vendeville A. Winzer K. Heurlier K. Tang C. M. Hardie K. R. (2005). Making “sense” of metabolism: autoinducer-2, LUXS and pathogenic bacteria. *Nat. Rev. Microbiol.* 3 383–396. 10.1038/nrmicro1146 15864263

[B69] Wang J. Li L. Chai R. Zhang Z. Qiu H. Mao X. (2019). Succinyl-proteome profiling of *Pyricularia oryzae*, a devastating phytopathogenic fungus that causes rice blast disease. *Sci. Rep.* 9:3490. 10.1038/s41598-018-36852-9 30837482 PMC6401317

[B70] Wang Y. Guo Y. R. Liu K. Yin Z. Liu R. Xia Y. (2017a). KAT2A coupled with the α-KGDH complex acts as a histone H3 succinyltransferase. *Nature* 552 273–277. 10.1038/nature25003 29211711 PMC5841452

[B71] Wang Y. Zhang L. Yu J. Huang S. Wang Z. Chun K. A. (2017b). A Co-drug of butyric acid derived from fermentation metabolites of the human skin microbiome stimulates adipogenic differentiation of adipose-derived stem cells: implications in tissue augmentation. *J. Invest. Dermatol.* 137 46–56. 10.1016/j.jid.2016.07.030 27498050

[B72] Weinert B. T. Schölz C. Wagner S. A. Iesmantavicius V. Su D. Daniel J. A. (2013). Lysine Succinylation is a frequently occurring modification in prokaryotes and eukaryotes and extensively overlaps with Acetylation. *Cell Rep.* 4 842–851. 10.1016/j.celrep.2013.07.024 23954790

[B73] Williams M. R. Costa S. K. Zaramela L. S. Khalil S. Todd D. A. Winter H. L. (2019). Quorum sensing between bacterial species on the skin protects against epidermal injury in atopic dermatitis. *Sci. Transl. Med.* 11:eaat8329. 10.1126/scitranslmed.aat8329 31043573 PMC7106486

[B74] Wu L. Gong T. Zhou X. Zeng J. Huang R. Wu Y. (2019). Global analysis of lysine succinylome in the periodontal pathogen *Porphyromonas gingivalis*. *Mol. Oral Microbiol.* 34 74–83. 10.1111/omi.12255 30672658

[B75] Xie L. Liu W. Li Q. Chen S. Xu M. Huang Q. (2015). First Succinyl-proteome profiling of extensively drug-resistant *Mycobacterium tuberculosis* revealed involvement of Succinylation in cellular physiology. *J. Proteome Res.* 14 107–119. 10.1021/pr500859a 25363132

[B76] Xu H. Wang Y. Lin S. Deng W. Peng D. Cui Q. (2018). PTMD: a database of human disease-associated post-translational modifications. *Genom. Proteom. Bioinform.* 16 244–251. 10.1016/j.gpb.2018.06.004 30244175 PMC6205080

[B77] Xu L. Li H. Vuong C. Vadyvaloo V. Wang J. Yao Y. (2006). Role of the luxS quorum-sensing system in biofilm formation and virulence of *Staphylococcus epidermidis*. *Infect. Immun.* 74 488–496. 10.1128/IAI.74.1.488-496.2006 16369005 PMC1346618

[B78] Xu X. Liu T. Yang J. Chen L. Liu B. Wei C. (2017). The first succinylome profile of *Trichophyton rubrum* reveals lysine Succinylation on proteins involved in various key cellular processes. *BMC Genom.* 18:577. 10.1186/s12864-017-3977-y 28778155 PMC5545033

[B79] Yang M. Wang Y. Chen Y. Cheng Z. Gu J. Deng J. (2015). Succinylome analysis reveals the involvement of lysine Succinylation in metabolism in pathogenic *Mycobacterium tuberculosis*. *Mol. Cell Proteom.* 14 796–811. 10.1074/mcp.M114.045922 25605462 PMC4390261

[B80] Yang X. Yu W. Shi L. Sun L. Liang J. Yi X. (2011). HAT4, a Golgi apparatus-anchored B-type histone acetyltransferase, acetylates free histone H4 and facilitates chromatin assembly. *Mol. Cell* 44 39–50. 10.1016/j.molcel.2011.07.032 21981917

[B81] Yao Z. Guo Z. Wang Y. Li W. Fu Y. Lin Y. (2019). Integrated Succinylome and metabolome profiling reveals crucial role of S-Ribosylhomocysteine lyase in quorum sensing and metabolism of *Aeromonas hydrophila*. *Mol. Cell Proteom.* 18 200–215. 10.1074/mcp.RA118.001035 PMC6356075 30352804

[B82] Yuan H. Chen J. Yang Y. Shen C. Xu D. Wang J. (2019). Quantitative succinyl-proteome profiling of Chinese hickory (*Carya cathayensis*) during the grafting process. *BMC Plant Biol.* 19:467. 10.1186/s12870-019-2072-8 31684873 PMC6829946

[B83] Zhang H. Li P. Ren S. Cheng Z. Zhao G. Zhao W. (2019). ScCobB2-mediated lysine desuccinylation regulates protein biosynthesis and carbon metabolism in *Streptomyces coelicolor*. *Mol. Cell Proteom.* 18 2003–2017. 10.1074/mcp.RA118.001298 PMC6773565 31337674

[B84] Zhang N. Jiang N. Zhang K. Zheng L. Zhang D. Sang X. (2020). Landscapes of protein posttranslational modifications of African *Trypanosoma* parasites. *iScience* 23:101074. 10.1016/j.isci.2020.101074 32403088 PMC7218301

[B85] Zhang Y. Wang G. Song L. Mu P. Wang S. Liang W. (2017). Global analysis of protein lysine Succinylation profiles in common wheat. *BMC Genom.* 18:309. 10.1186/s12864-017-3698-2 28427325 PMC5397794

[B86] Zhang Z. Tan M. Xie Z. Dai L. Chen Y. (2011). Identification of lysine Succinylation as a new post-translational modification. *Nat. Chem. Biol.* 14 58–63. 10.1038/nchembio.495 PMC3065206 21151122

[B87] Zhen S. Deng X. Wang J. Zhu G. Cao H. Yuan L. (2016). First comprehensive proteome analyses of lysine Acetylation and Succinylation in seedling leaves of *Brachypodium distachyon* L. *Sci. Rep.* 6:31576. 10.1038/srep31576 27515067 PMC4981852

